# Probe-Level Analysis of Expression Microarrays Characterizes Isoform-Specific Degradation during Mouse Oocyte Maturation

**DOI:** 10.1371/journal.pone.0007479

**Published:** 2009-10-16

**Authors:** Jesse Salisbury, Keith W. Hutchison, Karen Wigglesworth, John J. Eppig, Joel H. Graber

**Affiliations:** 1 Functional Genomics PhD Program, University of Maine, Orono, Maine, United States of America; 2 Department of Biochemistry, Microbiology and Molecular Biology, University of Maine, Orono, Maine, United States of America; 3 The Jackson Laboratory, Bar Harbor, Maine, United States of America; Victor Chang Cardiac Research Institute (VCCRI), Australia

## Abstract

**Background:**

Gene expression microarrays have provided many insights into changes in gene expression patterns between different tissue types, developmental stages, and disease states. Analyses of these data focused primarily measuring the relative abundance of transcripts of a gene, while treating most or all transcript isoforms as equivalent. Differences in the selection between transcript isoforms can, however, represent critical changes to either the protein product or the posttranscriptional regulation of the transcript. Novel analyses on existing microarray data provide fresh insights and new interpretations into transcriptome-wide changes in expression.

**Methodology:**

A probe-level analysis of existing gene expression arrays revealed differences in mRNA processing, primarily affecting the 3′-untranslated region. Working with the example of microarrays drawn from a transcriptionally silent period of mouse oocyte development, probe-level analysis (implemented here as *rmodel*) identified genes whose transcript isoforms have differing stabilities. Comparison of micorarrays measuring cDNA generated from oligo-dT and random primers revealed further differences in the polyadenylation status of some transcripts. Additional analysis provided evidence for sequence-targeted cleavage, including putative targeting sequences, as one mechanism of degradation for several hundred transcripts in the maturing oocyte.

**Conclusions:**

The capability of probe-level analysis to elicit novel findings from existing expression microarray data was demonstrated. The characterization of differences in stability between transcript isoforms in maturing mouse oocytes provided some mechanistic details of degradation. Similar analysis of existing archives of expression microarray data will likely provide similar discoveries.

## Introduction

### Gene expression microarrays and isoforms

The presence of alternative transcript isoforms can complicate the interpretation of gene expression microarray data [1], [2]. Microarrays commonly measure transcript expression through one or more short (25–60 nt) oligonucleotide probes that hybridize specifically to a gene of interest. In microarrays that use multiple short probes for each transcript, the probes are collectively referred to as a probeset, and the expression level reported for the gene is a summarization of the signal reported for each of the individual probes in the probeset [3]. If the probes that hybridize to a gene don't sample all isoforms equally, expression differences among isoforms can result in excessive variation in summarized probeset expression values. Accordingly, probeset re-mapping efforts in recent years have focused on identifying the unique probes that map to gene regions that are constitutively expressed across tissues and developmental stages [4]. These updated probesets have led to improved gene expression interpretation [4], [5], [6], however they can mask potentially critical changes in expression that are manifested as changes in isoform rather than total transcript abundance.

A majority of mammalian genes can be expressed as alternative isoforms [7], [8], including alternative splicing (AS), alternative transcription initiation, and alternative polyadenylation (APA). Isoform differences in the 3′-untranslated region (3′-UTR) are significant because 3′-UTRs are often home to post-transcriptional regulatory elements that control degradation, localization, and translation of the transcript. Transcriptome-wide truncation of 3′-UTR sequences has been identified in developing spermatocytes [9], proliferating cell lines [10], and cancer cell lines [11]. Conversely, a bias towards elongated 3′-UTRs has been found in ovulated oocytes and zygotes [12], developing embryos [13], and neurological tissues [14]. Ovaries were also shown to have a bias toward use of upstream APA sites [14], a feature consistent with the presence of a large number of transcripts with short 3′-UTR sequences that are degraded in the transition from *GV*-oocyte to 2-cell stage embryo [12]. Regulatory elements in the 3′-UTR are typically targets for miRNAs [15] or RNA-binding proteins [16]. MiRNAs can play multiple roles, suppressing translation directly or through targeting transcript for degradation through deadenylation or endonucleic cleavage [17].

Our fundamental hypothesis was that probe-level analysis of gene expression microarray data, especially the 3′-end targeted arrays associated with oligo-dT primed cDNA, would reveal changes in mRNA processing, including differences in polyadenylation and transcript stability.

### Measuring transcript degradation in the developing mammalian oocyte

The developing oocyte is transcriptionally silent [18] while over half of the total mRNA is degraded and/or deadenylated [19]. Changes in the mammalian maternal transcriptome during the transition from germinal vesicle (*GV*) to metaphase II (*MII*) arrested oocytes provide data that enable assessment of differences in transcript stability. Oocyte development requires transcript regulation by small RNAs as demonstrated by *Dicer* knockout experiments that reduce miRNAs in the *MII* oocyte, leading to arrested development and deregulation of mRNA expression profiles [20], [21]. Previous studies identified the genes whose transcripts are targeted for degradation [22], but largely ignored questions of differential stability among the isoforms of a single gene. We now use this large, defined perturbation of the transcriptome to demonstrate how probe-level analysis can reveal differences in stability and processing among isoforms. The analysis also serendipitously reveals details of processing in genes with only one isoform.

In this work, we used a probe-level analysis of Affymetrix Mouse GeneChip 430 version 2 (430v2) microarray data from *GV* and *MII* oocytes to identify differences in the stability among different transcript isoforms. The analysis was facilitated by a custom re-annotation of microarray probes focused on grouping together all probes that target transcripts from a single gene. Our analysis uses the change in expression at each probe rather than a summarized value for the probeset and identifies segmentations of the custom probesets where the *GV*-*MII* change in expression differs on either side of the segmentation point. Comparative analyses using *MII* microarrays that were hybridized with either random (*MII*) or oligo-dT (*MIIpa*) primed cDNA enabled differentiation between transcripts with and without polyA tails. The comparison revealed evidence of sequence-specific cleavage that left a protected, deadenylated 5′-fragment. Pattern analysis of the putative cleaved sequences revealed potential targeting patterns. Differential expression patterns for select genes were validated with quantitative reverse transcriptase PCR (qRT-PCR).

### Related work

Recent efforts have described the use of gene expression microarrays for investigation of differences in transcript isoforms. PLATA [10] performs analysis of Affymetrix Mouse Exon 1.0 ST arrays similar to that presented here, however the authors explicitly designed their Chip Definition File (CDF) for alternative UTRs based on putative known polyadenylation sites, rather than testing all possible segmentations. In contrast, *rmodel* tests all possible segmentations, allowing identification of novel processing events such as cleavage and 3′-UTR initiated transcription. In addition, the explicit comparison of oligo-dT and random primed cDNA from common samples enabled the investigation of the polyA status of transcripts. A more detailed comparison of *rmodel* and PLATA (Supplemental Text S1) revealed that despite the differences in procedural details, their results are largely equivalent when tests are performed on the same putative segmentations on the same microarray data. Several other approaches area also available targeted at Exon arrays [23], [24], [25]. FIRMAGene [25] was designed primarily for investigation of alternative splicing rather than polyadenylation, and application was only described for the newer Gene ST 1.0 chips, which use a random cDNA priming and probes spread throughout the transcript to assess transcript abundance. The coverage of the ST 1.0 gene chips in 3′-terminal exons, and especially in the 3′-UTR, is comparatively limited, making them less well suited to investigation of changes at the 3′-terminus than the earlier oligo-dT primed gene chips, *e.g.*, Affymetrix's Mouse GeneChip 430 version 2 (430v2) or Human GeneChip HU133 plus 2 (HU133p2).

## Results

### Extending gene annotations and generating custom probeset definitions

**Figure 1:**
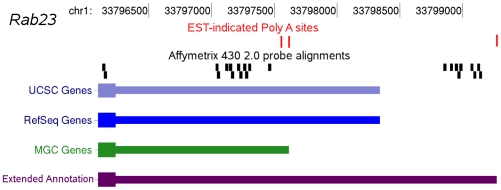
An example of an EST extended gene annotation is shown for Rab23. Available gene annotations for this gene do not extend to the distal 3′ polyadenylation site. By including this distal region in the extended gene annotation, an additional group of Affymetrix probes are included in the analysis.

We created a custom set of extended gene annotations using data with the goal of unifying all probes that target products of a given gene, regardless of isoform, into a single probeset. The extended gene annotations formed the basis for a custom CDF that was used in our probe-level microarray analysis. Transcript annotations from multiple sources (Methods) were pooled and extended using EST-indicated polyadenylation sites drawn from PACdb [26] (Figure 1) to produce 57,875 distinct transcripts. These distinct transcripts represent 26,021 non-redundant gene annotations, of which 14,513 (55%) do not match the annotated genomic coordinates found in the original tables, and represent novel annotations resulting from either inclusion of alternative exon sequence or extension of 5′ or 3′ UTRs. The original 430v2 probeset definitions provided multiple probesets for a single gene, typically targeting different isoforms, including mutually exclusive isoforms. Accordingly, the extended gene annotations do not necessarily reflect a logical transcription sequence for any given gene. Instead the new probesets enable comparison of expression levels within and among the resulting transcripts. Re-mapping and consolidation of the 496,468 mouse 430v2 array probe sequences to the NCBI build 37 mouse genome identified 403,718 unique probes (81% of total), of which 344,849 probes (69% of total) mapped to an exon or UTR in our extended gene annotations (Table 1).

**Table 1:** Probes used in redesigning probesets for Affymetrix arrays.

Array	total	aligned^a^	Distinct^b^	mRNA^c^	Probesets^d^
Affymetrix Mouse U74 v2	197 993	158 698	131 223	123 532	8 162
Affymetrix Mouse 430 v2	496 468	456 432	403 718	344 849	21 650
Affymetrix Human U95A v2	201 800	185 329	155 252	146 124	8 551
Affymetrix Human U133Av2	247 899	226 893	188 942	177 661	12 678
Affymetrix Human U133 Plus 2	604 258	562 248	484 344	393 924	23 581
Affymetrix Zebrafish	249 742	201 798	173 345	70 900	4 700

a number of probes successfully aligned to the current NCBI genomes with PASS [36].

b number of probes with a single match to the genome of the central 23 nucleotides with 1 or fewer mismatched nucleotide.

c number of distinct probes that mapped to an exon or UTR of our expanded gene annotation set.

d total number of probesets in our custom CDF.

### Probe level analysis delineates transcript degradation and deadenylation

**Figure 2:**
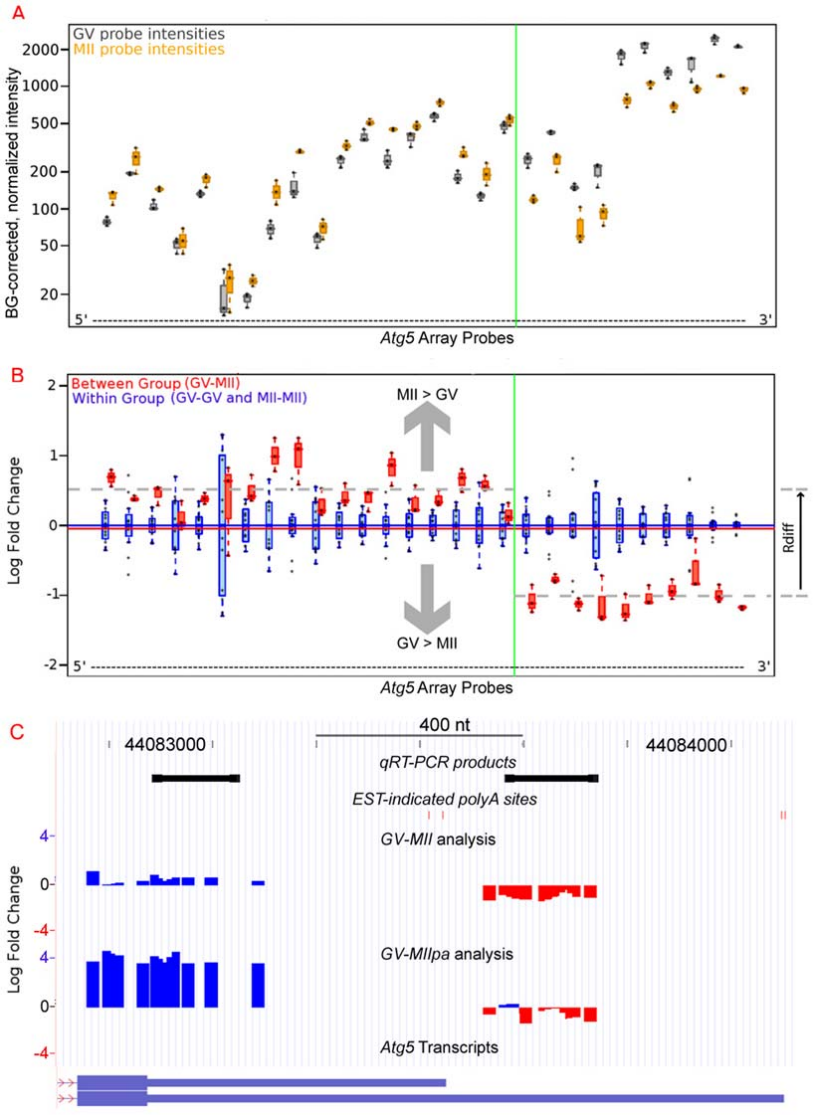
An ordered (5′ to 3′) boxplot of probe intensity comparisons indicates differential stability for isoforms of *Atg5* in the *GV*-*MII* transition. A. Background corrected and normalized probe intensities are shown for randomly primed cDNA from *GV* (gray) and randomly primed *MII* (orange) samples. The probe-specific differences in hybridization are typically constant among samples, unless portions of the gene are differentially expressed, as shown here for *Atg5*. B. The data from part A are transformed to a base2 logarithm of the *MII*-*GV* expression ratio for each probe (red box plots). For comparison, the plot includes the same measurement within biological replicates (blue box plots). A vertical green line indicates the optimum segmentation point for the *Atg5* probeset (determined with *rmodel*). The apparent increase in hybridization in the 5′-end of the probeset (inconsistent with transcriptional silence) is an artifact of normalization of the microarrays to a constant amount of RNA, and reflects the gain in abundance as a fraction of total RNA for a stable transcript. C. Display of the plot from part B on the UCSC Genome Browser [49] facilitates comparison of the *GV*-*MII* and *GV*-*MIIpa* analyses in conjunction with additional data. Transcript 3′-processing sites identified from ESTs are shown as red vertical bars near the top of each plot. Probes with increased expression at the *MII* stage (compared to *GV* stage) are shown in blue, whereas decreased expression is shown in red. Sequence target regions for qPCR validation are shown in black.

**Figure 3:**
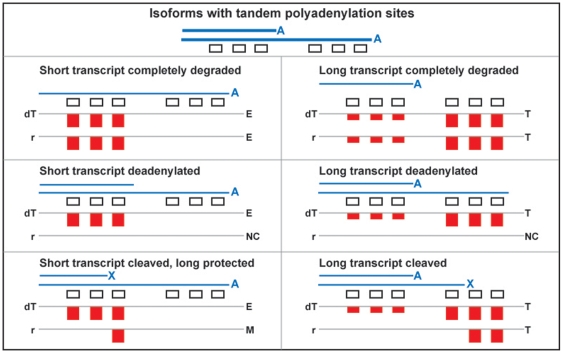
Predicted segmentation pattern signatures for various types of differential stability for isoforms that differ by use of tandem APA sites. Blue lines represent transcripts with tandem APA sites, in polyadenylated (A), deadenylated (no symbol), or cleaved (X) forms. The bar plots show predictions for the probe segmentation patterns of the log2-foldchange plots, as shown in Figure 2B. Predictions for additional conditions are available in Figure S1. Abbreviations: dT: oligo-dT primed *MII* oocyte cDNA compared to random primed *GV* oocyte cDNA; r: random primed *MII* oocyte cDNA compared to random primed *GV* oocyte cDNA; T: truncation; E: elongation; NC: no segmentation (uniform change); M: multiple events.

The *rmodel* package identifies processing events as segmentation points in a plot of the ratio of expression for two samples at each probe across the entire probeset (Figures 2 and 3). Processing events can encompass alternate generation of the transcript, *e.g.*, AS or APA, or subsequent processing, *e.g.*, degradation or deadenylation. *Rmodel* uses standard approaches to background correction and normalization (Figure 2A) [3], [27], [28], and finds segmentations in a plot of the base-2 logarithm of individual probe expression ratios (Figure 2B). Mapping the relative expression on the UCSC genome browser (Figure 2C) allows comparison with known genes. Segmentations are classified as a truncation when the downstream (3′) probes show a relative decrease in intensity ratio compared to upstream (5′), and elongation for the converse. In transcriptionally silent oocytes, truncation and elongation events are consistent with relative stabilization of short or long isoforms, respectively.

The well-characterized regulatory role of deadenylation and cytoplasmic polyadenylation in oocytes [29] led to the choice of cDNA generated with random primers for the *GV* and *MII* oocyte microarrays (referred to here as *GV* and *MII*, GEO Accession GSE5658 [22]). In contrast, the microarrays from the Dicer-knockout experiment (referred to as *MIIpa* and *MIIdko*) used the standard oligo-dT primed cDNA [20].

*Rmodel* can identify any type of change in mRNA processing, however the bias of 3′-end expression microarrays, combined with the general lack of introns in the 3′-UTR leads to a significant bias towards changes at the 3′-end of the resulting transcripts, which are expected to generate only one segmentation point in the probeset. Indeed, in the *GV*-*MII* comparison, we find (Table 2) that of 6289 probesets classified as expressed, 5230 (83%) display no segmentations. Of the remaining 1059, 878 (83%) have evidence for only a single segmentation, with 659 showing truncation and 219 elongation. The *GV*-*MIIpa* analysis shows similar bias towards single events (Table 3).

**Table 2:** Distribution of the number of genes with specific types of processing changes (events) in the probe-level comparison of GV and MII stage oocytes, using randomly primed GV oocyte cDNA and randomly primed MII oocyte cDNA (*GV-MII*).

	0 E^a^	1 E^a^	2 E^a^	Total
0 T^b^	5230	219	6	5455
1 T^b^	659	104	5	768
2 T^b^	43	14	4	61
3 T^b^	2	3	0	5
Total	5934	340	15	6289

a truncation;

b elongation.

**Table 3:** Distribution of the number of genes with specific types of processing changes (events) in the probe-level comparison of GV and MII stage oocytes, using randomly primed GV oocyte cDNA and oligo-dT primed cDNA (*GV-MIIpa*).

	0 E^a^	1 E^a^	2 E^a^	3 E^a^	4 E^a^	Total
0 T^b^	5338	596	36	3	0	5973
1 T^b^	638	171	36	1	1	847
2 T^b^	51	34	14	1	0	101
3 T^b^	1	0	3	2	1	7
Total	6028	801	90	7	2	6928

a truncation;

b elongation.

The comparison of *GV*-*MII* and *GV*-*MIIpa* analysis revealed significant differences in type of events (Table 4) that are likely a consequence of polyadenylation status, since transcripts lacking a polyA tail can be detected by random (*MII*), but not oligo-dT (*MIIpa*) priming. The data support three classes of changes between *GV* and *MII* oocytes: complete degradation, deadenylation, and cleavages that produce stable 5′-fragments. We can predict signatures in probeset segmentation patterns for the different cDNA priming for differences in stability of transcripts that differ only in use of tandem APA sites in a common terminal exon (Figure 3). Similar signatures can be predicted (Figure S1) for alternative terminal exons, 3′-UTR initiated transcripts [30], and genes with a single isoform that are subject to cleavage or deadenylation.

**Table 4:** A comparison of processing events identified in random and oligo-dT primed cDNA, where each cell represents the count of probesets that show specific counts of mRNA processing events in the *GV*-*MII* or *GV*-*MIIpa* analysis.

	NE^a^	NC^b^	1T^c^	1E^d^	M^e^	*GV-MII Total*
**NE** a	14429	754	35	28	2	15048
**NC** b	170	4003	413	416	228	5230
**1T** c	7	388	142	57	65	659
**1E** d	2	121	16	60	20	219
**M** e	1	72	32	35	41	181
***GV-MIIpa*** ** Total**	14409	5338	638	596	356	21337

Rows represent events in the *GV-MII* comparison; columns represent events in the *GV-MIIpa* comparison.

a **NE**: Not expressed in either tissue;

b **NC**: No processing difference identified (uniform);

c **1T**: 1 truncation identified;

d **1E**: 1 elongation identified;

e **M**: multiple events identified.

Complete degradation of a long isoform leads to truncation at a common site in the *GV*-*MII* and *GV*-*MIIpa* analyses, such as was observed in the probeset for Autophagy-related 5 (*Atg5*, MGI:1277186) transcripts (Figure 2). Similarly, complete degradation of a short isoform leads to elongation at a common site in both *GV*-*MII* and *GV*-*MIIpa* analyses, seen in the probeset for transcripts of *Dicer1* (MGI:2177178, Figure S2). 91 probesets matched this pattern for degradation of the short isoform, while 132 indicated degradation of the long isoform.

Deadenylation of a transcript prevents detection with oligo-dT primers, but not random primers. When multiple isoforms are present and differentially deadenylated, the expected pattern is segmentation of the *GV*-*MIIpa* relative expression plot, but no change in the *GV*-*MII* plot (Figure 3). These data cannot distinguish between deadenylation and cleavage (described below) between the polyA tail and the closest hybridization probe, however deadenylation and cleavage are grouped separately due to their different signatures. Deadenylation of a short isoform (e.g., *Ppap2b*, MGI:1915166, Figure S3) results in detection of an elongation in the *GV*-*MIIpa* analysis. Conversely, deadenylation of a long isoform (e.g., *Rdh11*, MGI:102581, Figure S4) results in truncation in the *GV*-*MIIpa* analysis. 448 probesets matched the pattern for deadenylation of the long isoform, while 444 indicated deadenylation of the short isoform (Table 4).

**Figure 4:**
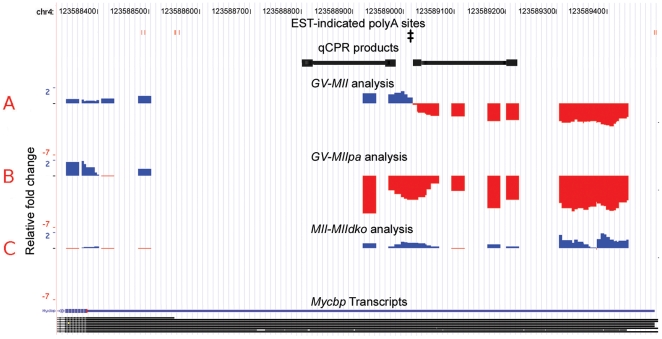
Mycbp has evidence of a protected short isoform and sequence specific cleavage of the long isoform. Probes with increased hybridization signal at the *MII* stage (compared to *GV* stage) are shown in blue, decreased expression is shown in red. Putative transcript 3′-processing sites identified from ESTs are shown as red vertical bars near the top of each plot. Sequence target regions for qPCR validation are shown in black. The location matched by motif 1, as identified by Gibbs Sampling (Figure 5), is indicated by (‡). A. The *GV*-*MII* analysis indicates a specific location of a transcript processing event for the *Mycbp* gene. B. The *GV*-*MIIpa* comparison segmentation coincides with the polyA site of the short isoform, indicating relative loss of the long isoform. Oligo-dT priming cannot amplify the extended fragment apparent in part (A). C. The *MIIpa*-*MIIdko* analysis shows that loss of *Dicer* activity partially restores signal from extended 3′-UTR (approximately 1.5 bits).

Cleavage of transcripts resulting in a protected 5′-fragment is indicated by truncation in the *GV*-*MII* analysis, without a corresponding segmentation in the *GV*-*MIIpa* analysis. The simplest cases to interpret are single truncations in the *GV*-*MII* analysis, and either no expression or no segmentation in the *GV*-*MIIpa* analysis. 395 probesets matched this pattern, including the probeset targeted to transcripts of *Ehf* (MGI:1270840, Figure S5). Cleavages are also indicated in 127 probesets with single truncations in both *GV*-*MII* and *GV*-*MIIpa* analyses, but at different positions. The probeset for *Myc* binding protein (*Mycbp*, MGI:1891750) transcripts provides an example of this phenomenon (Figure 4). The truncation indicated by the *GV*-*MIIpa* analysis aligns well with the known alternative transcript, and further indicates that the shorter transcript is stable and retains a polyA tail. The *GV*-*MII* array comparison indicated a truncation further downstream at a specific narrow region that has neither EST-evidence of a 3′-processing site nor recognizable polyA signals. The *MIIpa*-*MIIdko* comparison supports miRNA processing of the longer *Mycbp* transcript, as the Dicer knockout partially stabilizes only the longer transcript (1.5 bits) when compared to wildtype *MII* oocytes (Figure 4).

Additional patterns can be identified, but require more complex models for interpretation. For example, 60 probesets were characterized with single elongation events in both the *GV*-*MII* and *GV*-*MIIpa* analysis, including the probeset for transcripts of *Arf6* (MGI:99435, Figure S6). *Arf6* transcripts have a 3′-terminal exon with EST evidence of three distinct clusters of polyadenylation sites. The *GV*-*MII* elongation site matches the first polyA site, whereas the *GV*-*MIIpa* elongation matches the second polyA site. This combination of events supports a model where transcripts that terminate at the first polyA site are completely degraded, transcripts that terminate at the second polyA site are deadenylated, and full-length transcripts are stable. Such complex events defy the easy classification shown above for probesets with only a single event, however, visualization on a genome browser with additional data, such as known transcripts and ESTs, can facilitate interpretation on a gene-by-gene bases. Complete data for all the *GV-MII* and *GV-MIIpa* comparisons are available as a supplement (http://harlequin.jax.org/rmodel/).

### Sequence analysis of putative cleaved transcripts identifies target patterns

**Figure 5:**
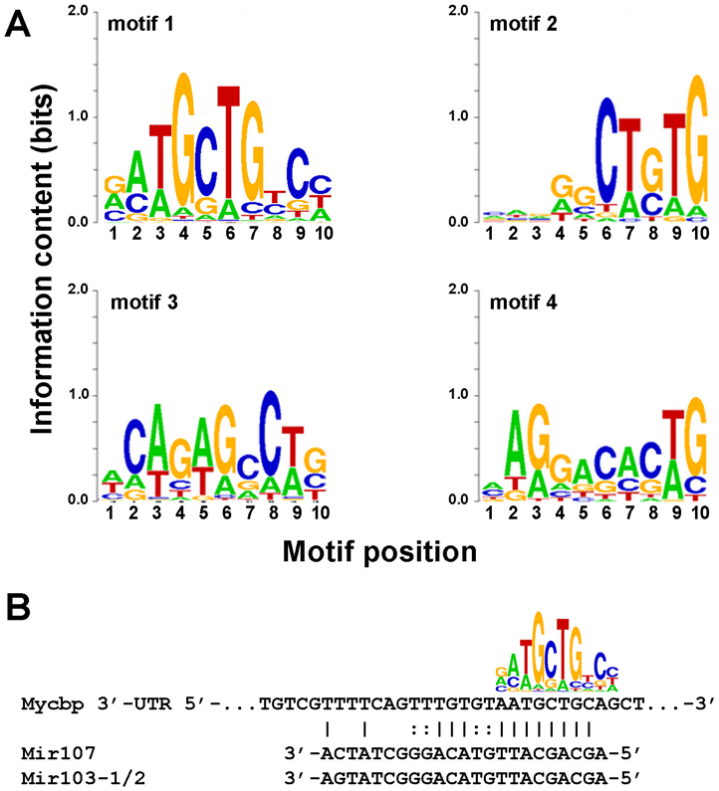
A sampling of representative motifs from Gibbs Sampling analysis of the sequence between probes that are separated by apparent cleavage sites. A. Sequence Logo [50] representations of four representative motifs identified by the Gibbs Sampler [51]. Motif 1 came from the first pass analysis, while motifs 2–4 came from the second pass analysis (Methods). Additional motifs are available in Figure S7. B. A match of motif 1 to the *Mycbp* 3′-UTR, also showing putative targe sites for Mir107 and Mir103-1/2, as identified by Miranda [34].

Sequence fragments from the segmentation regions of transcripts classified as cleaved were analyzed with the Gibbs Sequence Sampler [31] and revealed several prominent sequence motifs (Figure 5A; Additional results are available in Figure S7).

A survey of miRBase [32] showed that the motif 1 in Figure 5A is consistent with the miRNA seed regions (reviewed in [33]) of Mir107 and Mir103-1/2. Both of these miRNAs have evidence of expression in mouse oocytes [20], [21]. Intriguingly, a strong match to motif 1 is located in the correct in the narrow window identified for cleavage of the *Mycbp* transcript (Figure 5B). Sequence scanning of this region with miRanda [34] identified the motif position as a target for Mir103-1/2 (MGI:3619058 and MGI:3619059) and Mir107 (MGI:3619063) miRNAs with binding energies −21.4 and −20.9 kCal/mol, respectively (Predicted alignments are shown in Figure 5B).

### qRT-PCR validates cleavage/degradation events

**Figure 6:**
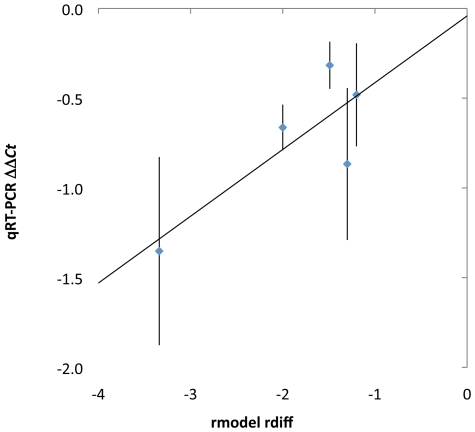
Changes in the proportion of transcripts identified by microarray analysis were validated by qRT-PCR. Microarray analysis is plotted as the *rdiff* score (Figure 2B). qRT-PCR results are plotted analogously, as ΔΔ*Ct* values, the difference between the Δ*Ct* values (comparing *GV* and *MII* oocyte samples) in the products that flank the *rmodel*-predicted segmentation points. A linear fit of the data is shown. Error bars represent the standard error of three replicate qRT-PCR experiments.

Quantitative RT-PCR validated the probeset segmentation patterns identified by the *GV*-*MII* comparisons for *Atg5*, *Cnot2*, *Baiap2l1*, *G6pdx* and *Mycbp* transcripts (Figure 6), a group of genes specifically chosen to validate the truncations that were and were not consistent with known APA sites.

Microarray predictions are presented as rdiff scores (illustrated in Figure 2), representing the difference in log2-ratio of signal upstream and downstream of the at the segmentation point. qRT-PCR results are presented in an analogous manner. The change in each portion of the transcript during the *GV*-*MII* transition is measured with a qRT-PCR product, and the difference in the threshold cycle (Δ*Ct*) value is analogous to the log2 ratio of expression. The qRT-PCR change within each transcript is therefore calculated as ΔΔ*Ct*, the difference in the Δ*Ct* values between the upstream and downstream products. As shown (Figure 6) the qRT-PCR values correlated well in both the classification of processing (truncation) and magnitude of the difference between expression levels in the 5′- and 3′-portions of the transcript.

The genomic regions indicated by the probeset segmentations for *Atg5* and *Cnot2* are consistent with EST-supported APA sites [26], while those for *Baiap2l1*, *G6pdx* and *Mycbp* have no such evidence. The lack of EST evidence for alternative 3′-processing and the absence of polyA signal and downstream element sequences are consistent with transcript cleavage events in which the 5′-most region is more stable than the 3′-end [35]. Genomic coordinates and *rmodel* analysis for *Cnot2*, *Baiap2l1*, *G6pdx* are available in Figures S8, S9, and S10, respectively.

## Discussion

Canonical approaches to microarray analysis have been driven by the question of differential gene expression as measured by total transcripts encoded by a given gene. This focus has ultimately led to a focus on probes that target constitutive portions of transcripts [4], limiting the exploration of posttranscriptional regulation and/or selection of alternative isoforms. Although the signature of alternative isoforms is present in microarray experiments [1], [2], it may be overlooked in the standard summarization analysis of probes in constitutive regions of a gene. Ideally, a complete gene expression analysis would investigate change in both the total transcript level and in the relative abundance of variant isoforms.

We developed and used an extended set of gene annotations in conjunction with a probe-level microarray analysis program to detect the differential regulation of transcript isoforms. Given that *GV* and *MII* oocytes are transcriptionally silent, the results presented here focus on the posttranscriptional fate of the existing mRNA during the *GV*-*MII* oocyte transition. We found that microarray cDNA preparation methods have a profound impact on this analysis, in particular demonstrating that the comparison of microarrays hybridized to random-primed and oligo-dT-primed cDNA enabled the distinction between transcripts that were degraded, deadenylated, or cleaved in a sequence specific manner. Pattern analysis of cleavage events indicated putative targeting sequences during the *GV*-*MII* transition. Our investigation of the putative cleavage regions (*e.g.*, for the *Mycbp* transcript) indicates strong evidence of miRNA directed transcript cleavage.

The number of genes identified here with differential stability among transcript isoforms in the *GV*-*MII* transition is likely an underestimate. First, the threshold values set for acceptance of segmentation points were conservative (FDR<0.06 based on variation within replicates), a choice explicitly made to minimize false positive results. The ability to identify difference in transcript isoforms is also explicitly dependent up on the probe coverage on each individual gene. Our method also required that at least three probes be present on each site of a segmentation point. Finally, hybridization probes for the 430v2 were designed based on the available transcript data at the time, however, new data sets and improved technologies (e.g., [8], [36], [37]) have revealed additional, often extended isoforms not covered by existing probes. Indeed, updated transcript data for *Mycbp* (NCBI accession numbers AK132198 and AK037661 [36]) indicates additional extended transcript isoforms covering a few thousand nucleotides beyond the range covered on the 430v2.

Our work highlights the critical role that the method of cDNA priming can play in determining what transcripts and processing activities can be measured. A recent report utilized a similar microarray analysis to compare 3′-UTR characteristics in proliferating and non-proliferating cells [10]. The Mouse Exon 1.0 ST array protocols include cDNA generation with random primers and cannot distinguish between transcripts with and without polyA tails, which results in a common microarray pattern for polyadenylation at an upstream site and cleavage that produces a protected 5′-fragment without a polyA tail. Further experimental analysis will be required to differentiate between these interesting alternatives.

While new methods of transcript measurement are rapidly becoming available [37], [38], the usefulness of microarrays in the study of qualitative transcript biology still has not been fully explored. New algorithms such as *rmodel* may be applied to both novel experiments and retrospectively to existing microarray experiments. The public repository Gene Expression Omnibus [39] contains tens of thousands of 3′-end targeted expression microarray datasets (Table 5), many of which have been analyzed only for assessment of transcript abundance. Revisiting these data has the potential to provide new insights into mRNA processing under multiple conditions, while also guiding the choice of tissues and conditions for new investigations. In addition, since we focus on changes in signal in different portions of the transcript, the analytic approaches presented here should be adaptable to new data types, *e.g.*, mRNA-seq [8], [37].

**Table 5:** Count of GEO entries for a selection of 3′-targeted microarrays.

Platform	Count
Affymetrix GeneChip® Mouse Genome 430 2.0 Array	11 568
Affymetrix U74A version 2	5 599
Affymetrix Human Genome U133 Plus 2.0	26 067
Affymetrix Human Genome U133A	21 454
Affymetrix Human Genome U133B	4 694

The different transcript isoforms of a gene can exhibit significant differences in function and regulation, even when the final protein product is the same. Complete description of gene expression accordingly requires delineation of the distribution among isoforms along with total abundance of the transcript. Existing databases contain much data to address studies of differences in isoform expression, provided the proper tools are available. Probe-level analysis of gene expression microarray data (shown here with *rmodel*) has the capability to reveal previously hidden details of transcript isoform usage.

The *rmodel* source code and extended gene annotations for 11 microarray platforms are available at http://harlequin.jax.org/rmodel. Additional platforms will be added as resources become available.

## Methods

### Extending gene annotations

Merged, expanded gene annotations were generated from UCSC's knownGene [40] RefSeq [41], MGI' representative transcripts [42], GenBank's mRNA collection [43] and MGC gene [44], as extracted from the UCSC genome browser tables [45]. Putative 3′-terminal exons from these genomic projections were extended downstream into intronic or intergenic regions if there was EST evidence of extended UTRs in PACdb [26]. Extensions of gene annotations were not permitted to extend beyond the most 3′ transcription stop site plus 5000 nt or into the next 3′ annotated gene.

### Custom probeset generation

Probe sequences were obtained from the manufacturer's web site http://www.affymetrix.com. In order to asses uniqueness, all probes were aligned to the mouse C57BL6/J genome build NCBI Build 37 using PASS [46], as it provided the best tradeoff of speed and alignment sensitivity, especially for the analysis of near matches. Under the assumption that mismatches near the end of the probe are most likely to result in cross-hybridization, the central 23 nt of each probe was aligned, allowing a single base mismatch. Probes that matched more than one location in the genome were removed. Probes likely to be part of a mature mRNA were selected based on the expanded gene annotations.

### Microarray data

Microarray data files for *GV* and *MII* datasets [22] were obtained from the Gene Expression Omnibus [39] (Accession GSE5668). Oligo-dT primed array data files for the *MIIpa* and *MIIdko* datasets were generously provided by Richard M. Schultz [20].

### Identification of differences in mRNA processing with *rmodel*

Intensity measures from all chips were background corrected and normalized using standard methods [3], [27], [28]. Since normalized probe intensities still display probe-specific effects (Figure 2A), we compare each individual probe directly between arrays, working with the logarithm of the ratio of the normalized intensity (bit scores) for each probe (Figure 2B).

*Rmodel* was developed as a package for the open-source R-project. *Rmodel* divides a probeset into segments that represent the sequence boundaries of transcribed regions that change by different amounts when comparing two samples, as expected for alternative transcript processing events. Processing events can reflect changes in generation (*e.g.*, APA or AS) or subsequent processing (*e.g.*, degradation or deadenylation) of transcript isoforms. To identify segmentation patterns, *rmodel* considers all possible subdivisions of a probeset. Subdivisions are evaluated by walking along each probeset in a 5′ to 3′ direction, evaluating six probes at a time. A modified *t-*test is calculated from the three probes on either side of the segmentation point, using the median values of the three replicates for each probe in each sample. An additional condition was placed on the difference in logratios (*rdiff*) between the two sides of the segmentation.

All events reported in this paper are restricted to thresholds of |*t*-value|≥5.5. To reduce the incidence of false positives that arise through spuriously low variance in multiple testing [47], segmentation points were accepted only if |*rdiff*|≥1.0. In addition, probes were eliminated from consideration if the average background-corrected normalized intensity was not greater than 100 in at least one of the samples.

False Discovery Rates (FDR [48]) values were estimated as the ratio of above-threshold segmentations in a null model to above-threshold segmentations in the true distribution. Two null models were investigated. The first null model was generated through analysis of the microarray samples with randomization of the order of the probes within the customized probesets. An additional null model was tested using comparisons between replicate arrays rather than between the samples, without randomization of the probeset order. The estimated FDR for the *GV*-*MII* was 0.03 using the between replicate null model and 0.33 using the between sample null model. The estimated FDR for the *GV*-*MII* was 0.06 using the between replicate null model and 0.41 using the between sample null model.

### Quantitative RT-PCR validation

Quantitative RT-PCR analysis was used to confirm five segmentations identified in the *GV*-*MII* comparison. We limited our scope to highly expressed genes, both with and without EST-supported 3′-processing sites. Changes in the transcript isoform distribution were assayed by relative difference (*rdiff*) in threshold cycle scores between 5′ and 3′ qRT-PCR products.

Full grown oocytes (*GV*) were collected from 22d B6SJLF1 mice primed with 5 IUs PMSG (Calbiochem, cat 367222). *GV* samples were incubated in M199 w/5%fbs 18 hrs to develop M-phase oocytes (*MII*). Triplicate groups of 20 *GV* and *MII* oocyte mRNA was extracted with PicoPure columns (Arcturus, cat. KIT0202) according to the protocol for use with CapSure HS LCM Caps. Extraction protocol was modified to begin with entry step B1d and use 100 ul extraction buffer and ethanol precipitation volumes. Luciferase spike-in RNA (Promega Cat. L4561) was added as a carrier at the extraction buffer step (500 ng per reaction) to prevent loss of mRNA. Optional on column DNase treatment was incorporated as described in the PicoPure protocol Appendix A using a DNAse set from Qiagen (cat. 79254). Extracted RNA was immediately used in SuperScript III reverse transcription reaction (invitrogen, part no. 18080.pps) using random primers (Promega, cat. c1181).

QuantitativePCR was accomplished using Promega PCR master mix with SYBR Green 1 (Invitrogen, cat S7563) and ROX dye as a reference. All samples were tested in triplicate. Two sets of primers for each gene were designed to produce products which flank the apparent transcript processing event identified by microarray analysis (primers used are listed in Table S1). Each qPCR reaction had the cDNA equivalent of 0.1 oocyte and was analyzed on a Stratogene mX4000. Initial PCR products were examined for correct size and quality by ethidium bromide stained gel electrophoresis. All *Ct*s ranged from 24–32, and post reaction SYBR green dissociation curves all had single product temperature distributions with *Tm*>75C.

A baseline control such as a house keeping gene or spike-in RNA is necessary when comparing separate samples. The large change in the oocyte transcriptome during the *GV-MII* transition led us to use the Luciferase carrier as an internal control rather than attempting to identify a stable endogenous housekeeping gene. In addition, the comparison of interest is between portions of the same transcript, rather than between different transcripts, making the principal need for a control verification of the conversion from RNA to cDNA and amplification. The Luciferase spike in RNA was added to the oocyte extract before RNA isolation, verifying and validating all steps from RNA isolation onward. The qRT-PCR results for the Luciferase RNA are consistent across all samples (Figure S11), with relatively low *Ct* threshold values, reflecting the dual nature of the Luciferase RNA as a spike in and as a RNA carrier. The Luciferase data confirmed that the RNA extraction, cDNA reaction and qRT-PCR were successful and consistent.

### Sequence analysis

Transcript sequences representing putative cleavage site regions were analyzed using the Gibbs Sequence sampler [31]. Cleavage site regions were defined as the sequence between probes that flanked the processing event, and varied in size depending on probe placement. To facilitate Gibbs Sampling runs the analysis was restricted to sequences longer than 60 bp and shorter than 200 bp and run in randomly selected groups of 200 sequences at a time with the following command line gibbs -PBernoulli 10 -C 0.01 -i 100 -k 100 -p 50 -S 25 -Y -F -x -r -n. Searches for weaker signals were made with an additional run of the Gibbs Sampler after “near optimal” matches from the first round of detection were masked in the input sequences. Scanning of miRNAs for the *Mycbp* was accomplished with miRanda software [34] using program defaults.

## Supporting Information

Figure S1 Expected relative hybridization signature patterns for various types of processing events. Abbreviations: dT: oligo-dT primed MII oocyte cDNA compared to random primed GV oocyte cDNA; r: random primed MII oocyte cDNA compared to random primed GV oocyte cDNA; T: truncation; E: elongation; NC: no segmentation (uniform change); M: multiple events.
(0.39 MB TIF)

Figure S2 UCSC Genome browser view of *Dicer1* (MGI:2177178), which shows common elongation segmentation points in the transcripts as identified by *GV-MII* and *GV-MIIpa* analyses, indicating degradation of the shorter isoform.
(1.74 MB TIF)

Figure S3 UCSC Genome browser view of *Ppap2b* (MGI:1915166), which shows a transcript truncation segmentation point in the *GV-MIIpa* analysis and no segmentation in the *GV-MIIpa* analysis, indicating deadenylation of the longer isoform.
(2.15 MB TIF)

Figure S4 UCSC Genome browser view of *Rdh11* (MGI:102581), which shows a transcript elongation segmentation point in the *GV-MIIpa* analysis and no segmentation in the *GV-MIIpa* analysis, indicating deadenylation of the shorter isoform.
(2.35 MB TIF)

Figure S5 UCSC Genome browser view of *Ehf* (MGI:1270840), which shows a transcript truncation in the *GV-MII* analysis and no segmentation in the *GV-MIIpa* analysis, indicating a cleavage site.
(1.96 MB TIF)

Figure S6 UCSC Genome browser view of *Arf6* (MGI:99435), which shows different transcript elongation segmentation points in the *GV-MII* and *GV-MIIpa* analyses, indicating degradation of the transcripts that end at the first polyA site and deadenylation of the transcripts that end at the second polyA site (compared to the full length transcript).
(2.07 MB TIF)

Figure S7 Representative motifs identified in Gibbs Sampler [2] analysis of the sequence regions that flank putative cleavage sites. (A) Sixteen first pass analyses, with a random selection of 200 sequences from the overall set. (B) Sixteen seond-pass analyses, in which motifs identified in the first pass were masked.
(1.23 MB TIF)

Figure S8 UCSC Genome Browser view of *Cnot2* (MGI:1919318), showing the location of the rmodel predicted processing event (green line) and the location of the qRT-PCR products used to validate the processing change.
(0.66 MB TIF)

Figure S9 UCSC Genome Browser view of *Baiap2l1* (MGI:1914148) showing the location of the rmodel predicted processing event (green line) and the location of the qRT-PCR products used to validate the processing change.
(0.72 MB TIF)

Figure S10 UCSC Genome Browser view of *G6pdx* (MGI:105979), showing the location of the rmodel predicted processing event (green line) and the location of the qRT-PCR products used to validate the processing change.
(0.82 MB TIF)

Figure S11 qRT-PCR results for the control Luciferase mRNA that was spiked into the oocyte cell extracts before RNA isolation and all subsequent steps. Bar heights represent the average *Ct* value obtained in three replicates of each sample. Error bars represent the standard error. The relatively low *Ct* value reflects the Luciferase transcript's dual role as carrier and control.
(0.17 MB TIF)

Table S1 Primers used in qRT-PCR validation of microarray results.
(0.03 MB DOC)

Text S1 (0.04 MB DOC)
