# Supplementary figures and images for: Probe-Level Analysis of Expression Microarrays Characterizes Isoform-Specific Degradation during Mouse Oocyte Maturation
Source: PLoS One. 2009 Oct 16;4(10):e7479. doi: 10.1371/journal.pone.0007479 (PMC2759528; doi:10.1371/journal.pone.0007479)

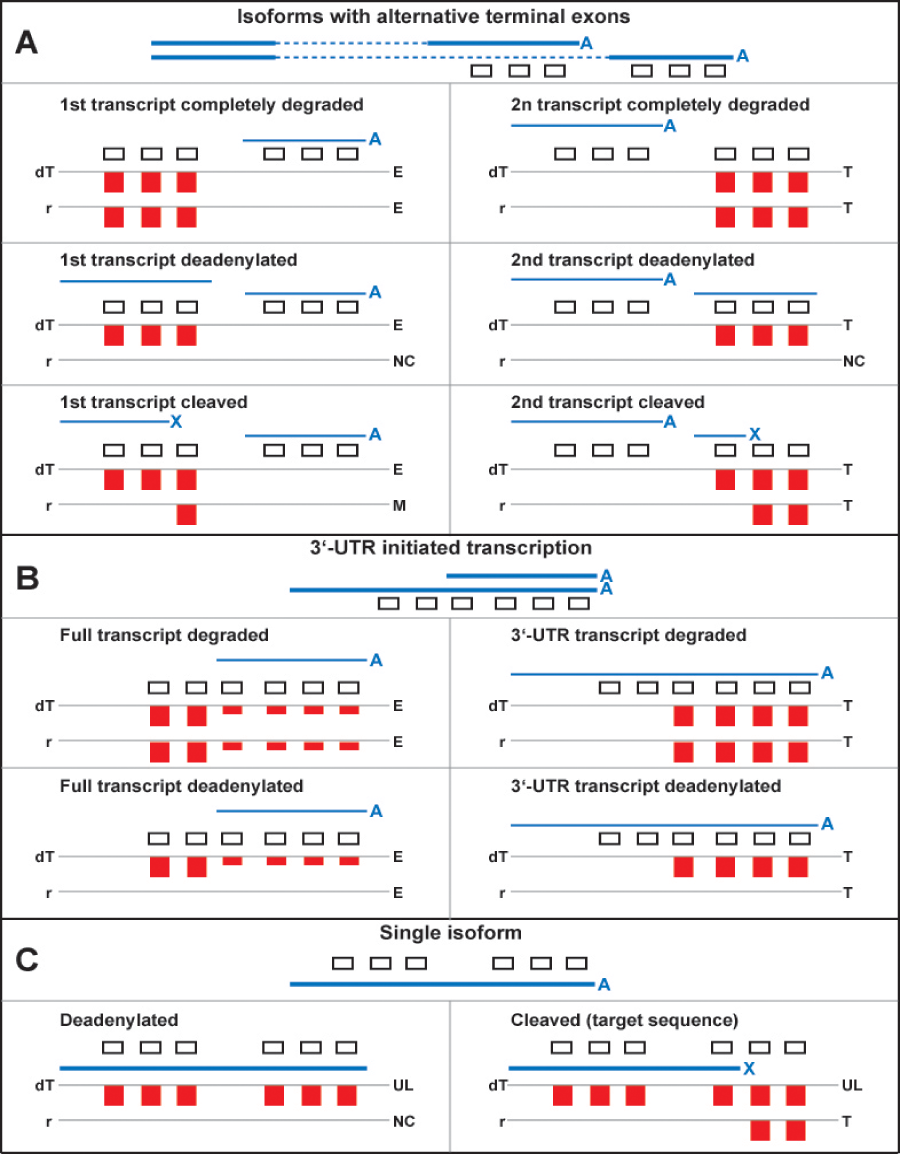

Supplement: Figure S1 — Expected relative hybridization signature patterns for various types of processing events. Abbreviations: dT: oligo-dT primed MII oocyte cDNA compared to random primed GV oocyte cDNA; r: random primed MII oocyte cDNA compared to random primed GV oocyte cDNA; T: truncation; E: elongation; NC: no segmentation (uniform change); M: multiple events. (0.39 MB TIF) [file pone.0007479.s001.tif]

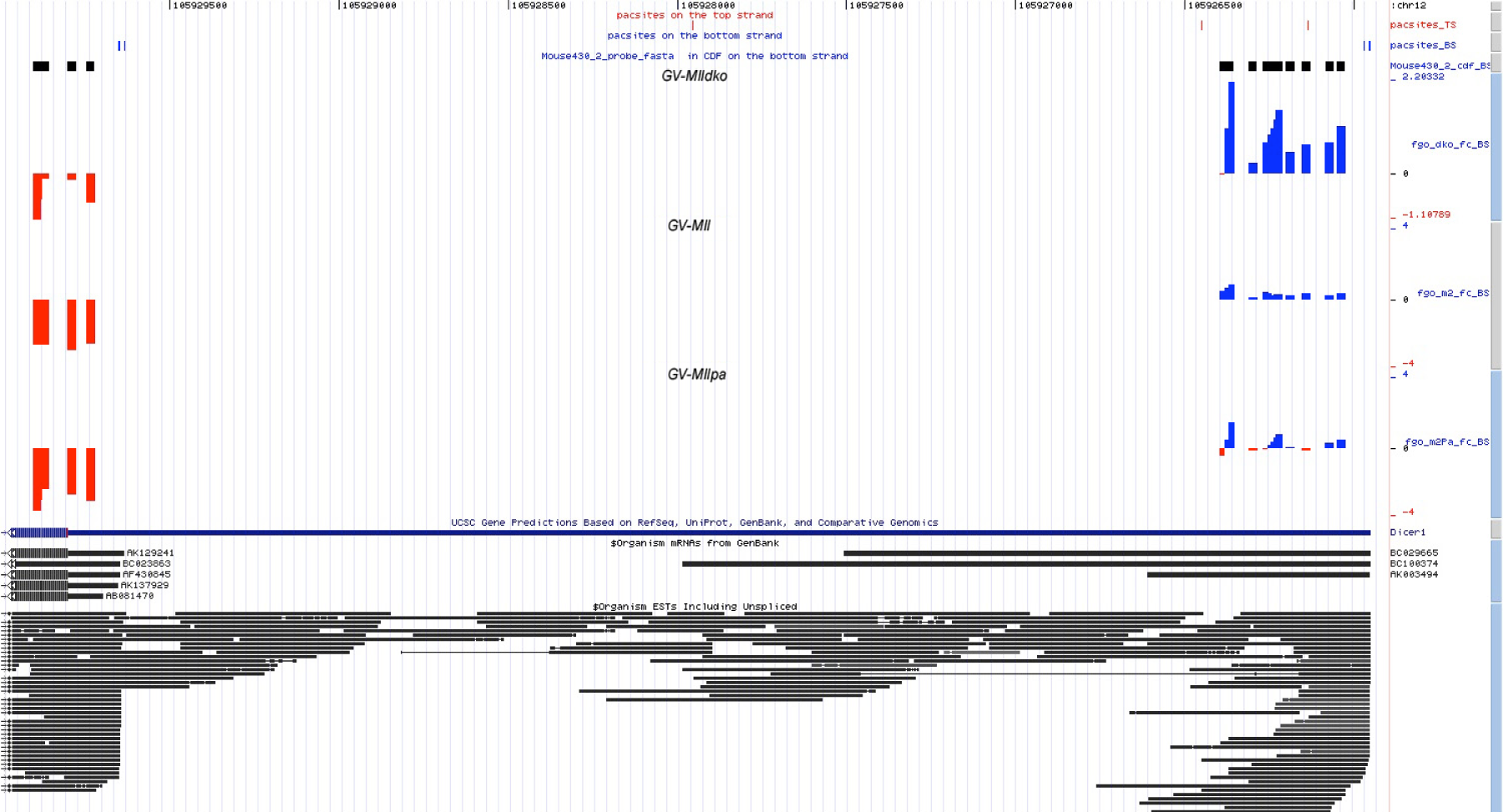

Supplement: Figure S2 — UCSC Genome browser view of Dicer1 (MGI:2177178), which shows common elongation segmentation points in the transcripts as identified by GV-MII and GV-MIIpa analyses, indicating degradation of the shorter isoform. (1.74 MB TIF) [file pone.0007479.s002.tif]

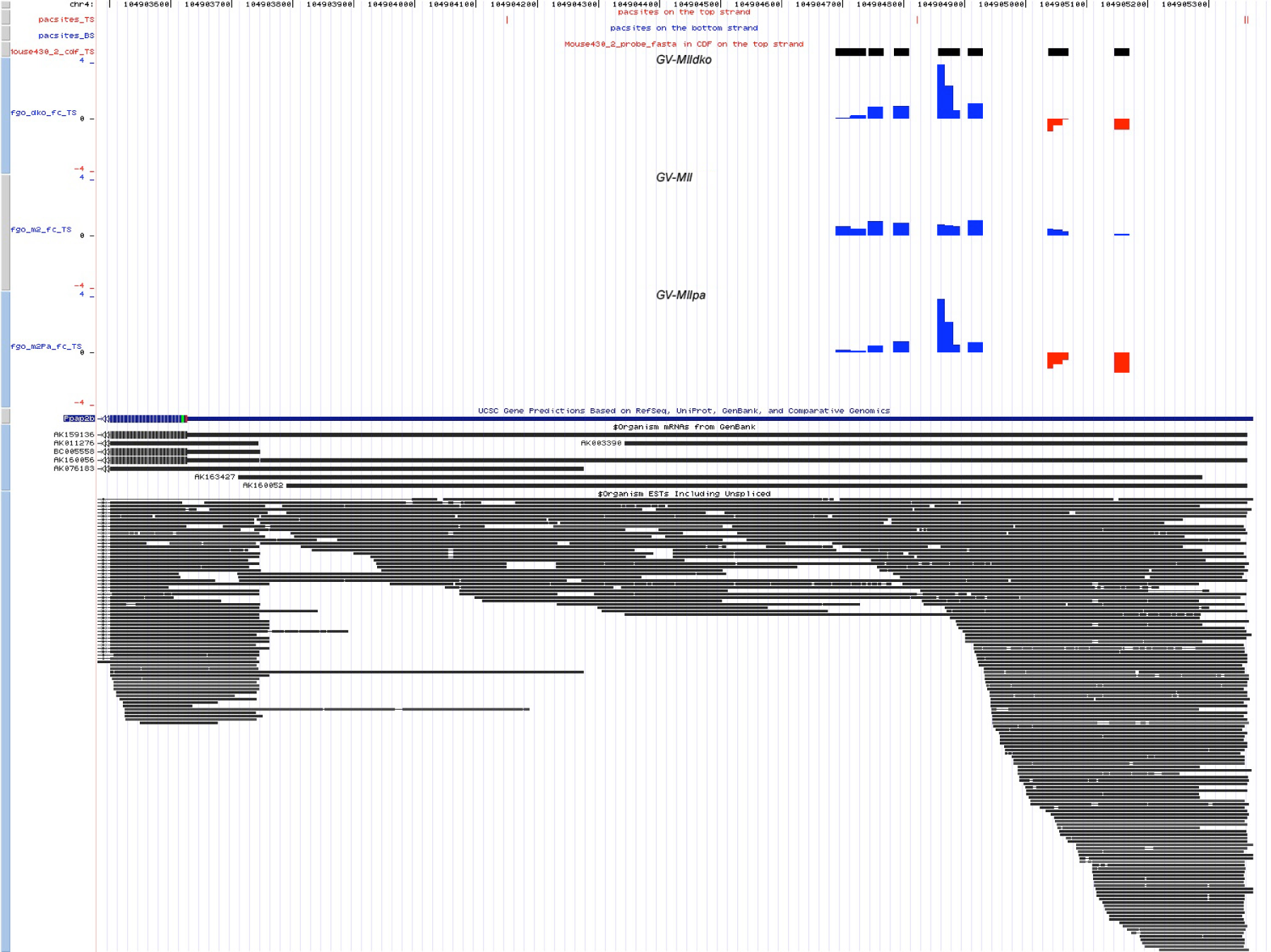

Supplement: Figure S3 — UCSC Genome browser view of Ppap2b (MGI:1915166), which shows a transcript truncation segmentation point in the GV-MIIpa analysis and no segmentation in the GV-MIIpa analysis, indicating deadenylation of the longer isoform. (2.15 MB TIF) [file pone.0007479.s003.tif]

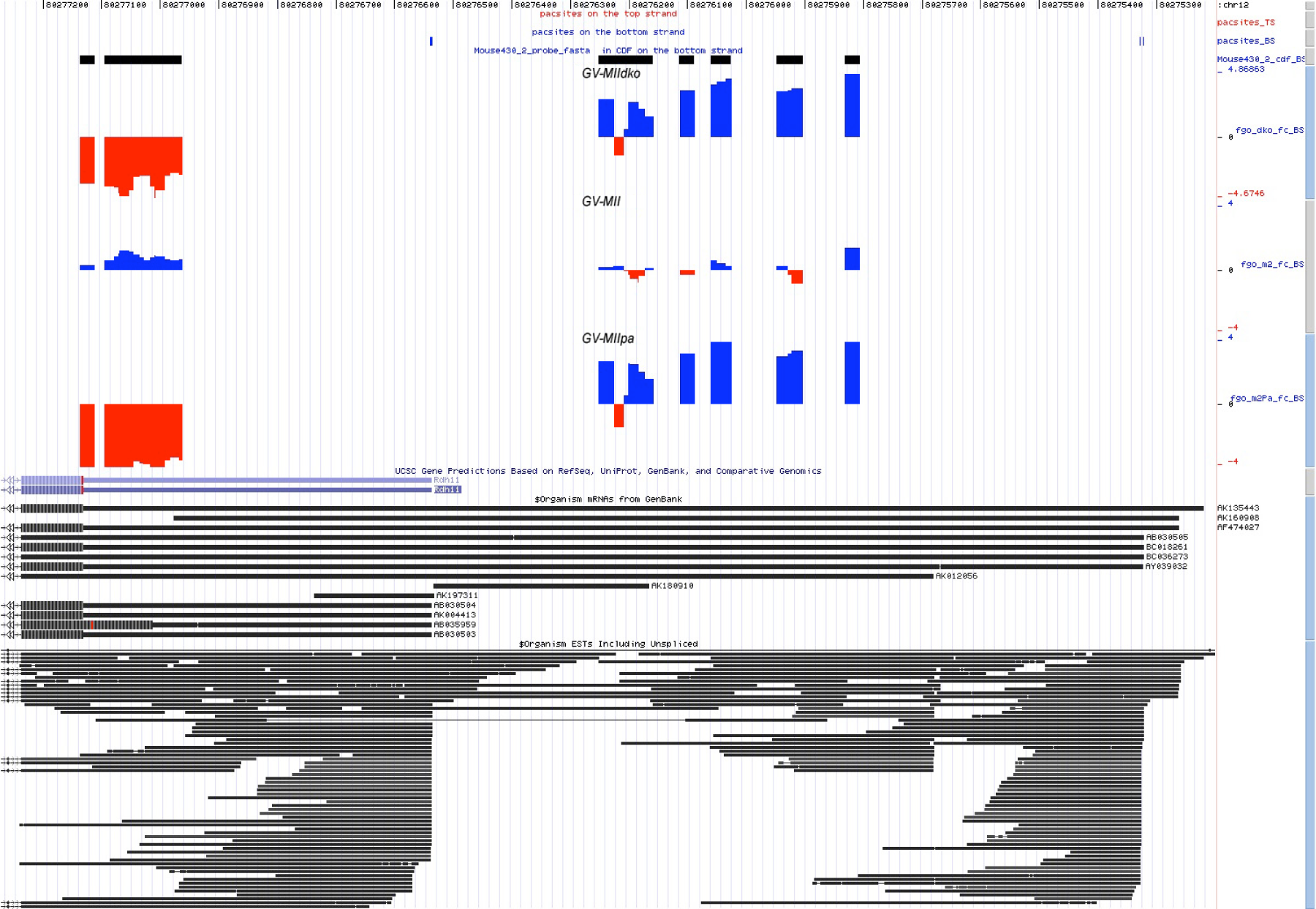

Supplement: Figure S4 — UCSC Genome browser view of Rdh11 (MGI:102581), which shows a transcript elongation segmentation point in the GV-MIIpa analysis and no segmentation in the GV-MIIpa analysis, indicating deadenylation of the shorter isoform. (2.35 MB TIF) [file pone.0007479.s004.tif]

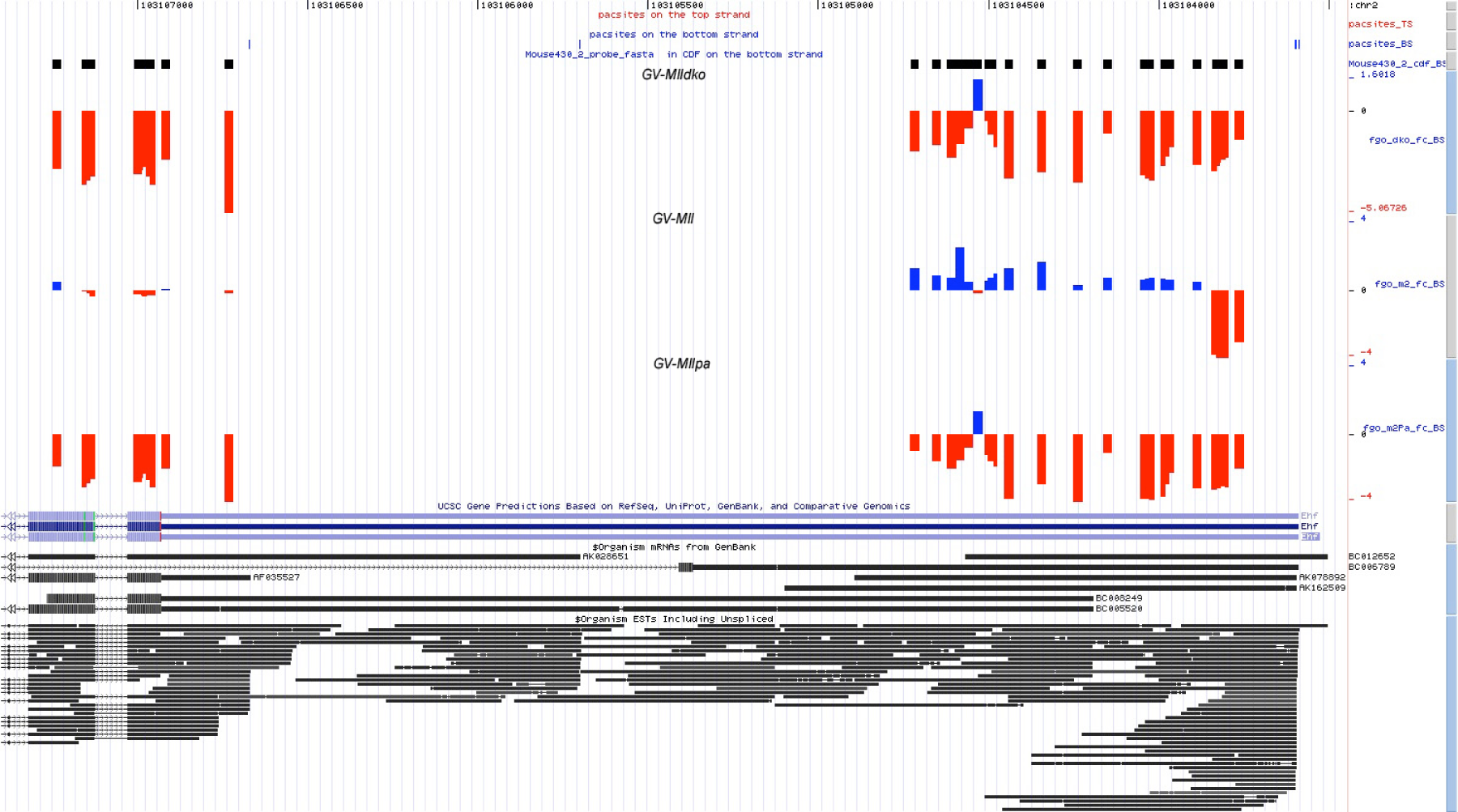

Supplement: Figure S5 — UCSC Genome browser view of Ehf (MGI:1270840), which shows a transcript truncation in the GV-MII analysis and no segmentation in the GV-MIIpa analysis, indicating a cleavage site. (1.96 MB TIF) [file pone.0007479.s005.tif]

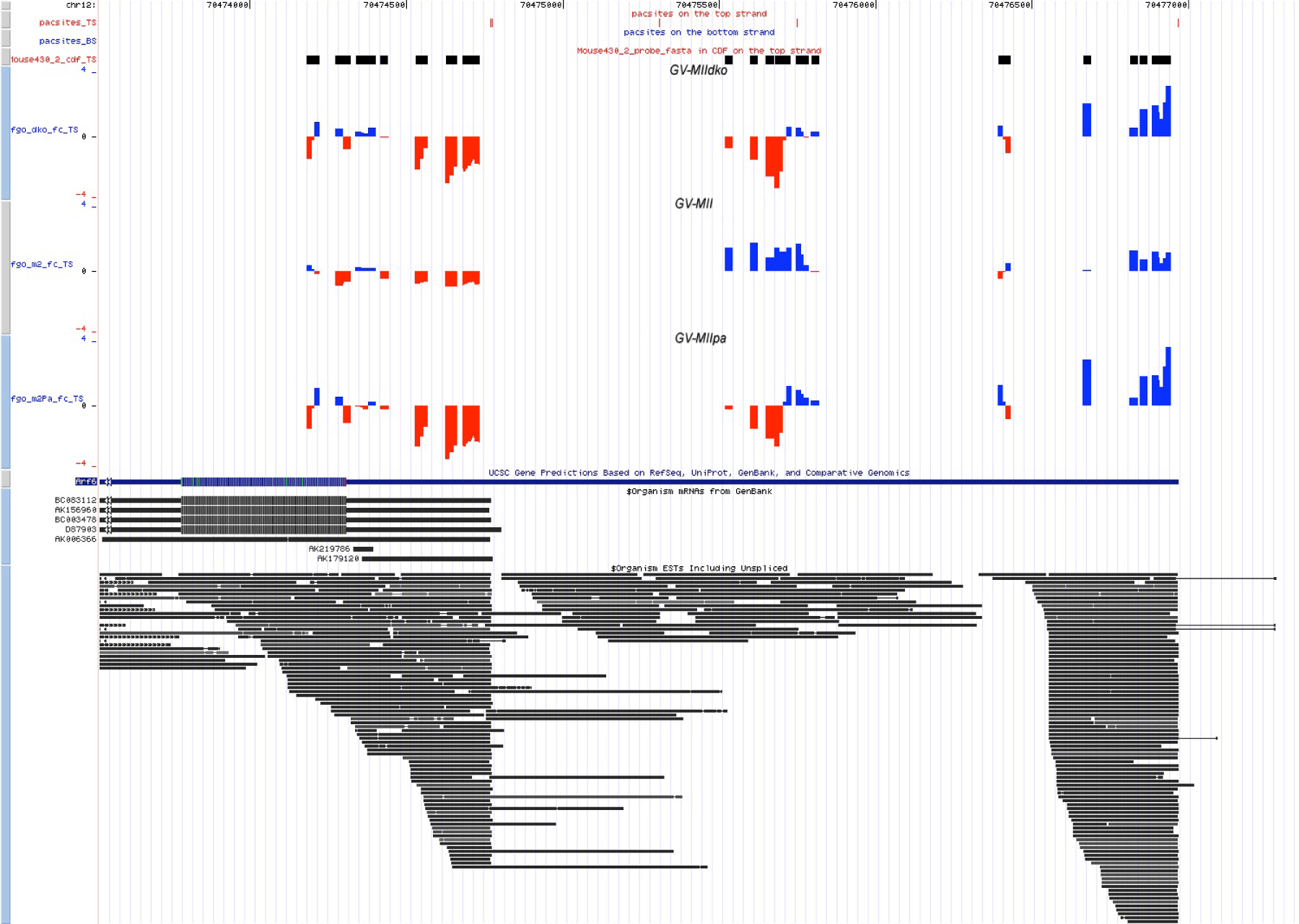

Supplement: Figure S6 — UCSC Genome browser view of Arf6 (MGI:99435), which shows different transcript elongation segmentation points in the GV-MII and GV-MIIpa analyses, indicating degradation of the transcripts that end at the first polyA site and deadenylation of the transcripts that end at the second polyA site (compared to the full length transcript). (2.07 MB TIF) [file pone.0007479.s006.tif]

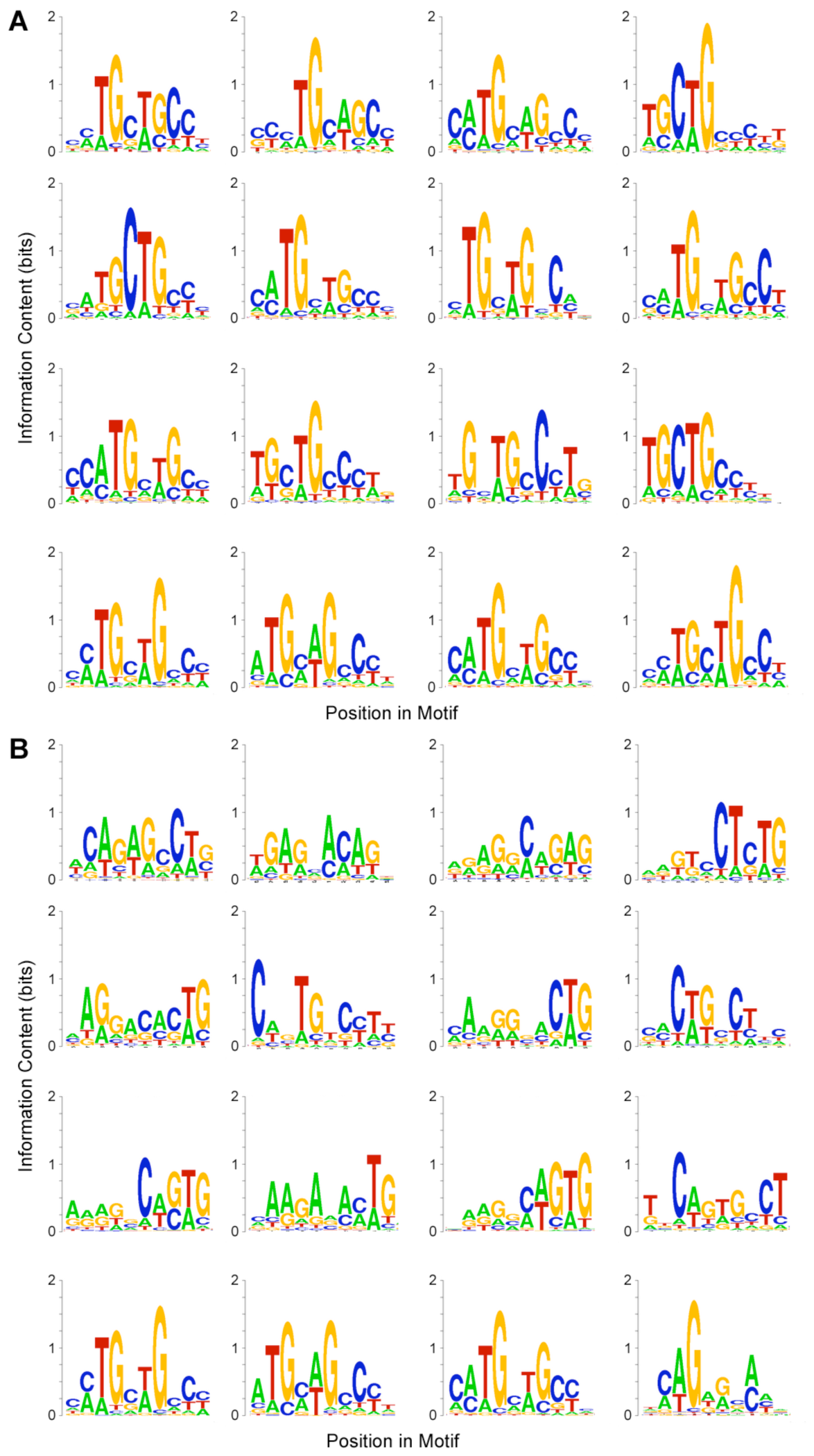

Supplement: Figure S7 — Representative motifs identified in Gibbs Sampler [2] analysis of the sequence regions that flank putative cleavage sites. (A) Sixteen first pass analyses, with a random selection of 200 sequences from the overall set. (B) Sixteen seond-pass analyses, in which motifs identified in the first pass were masked. (1.23 MB TIF) [file pone.0007479.s007.tif]

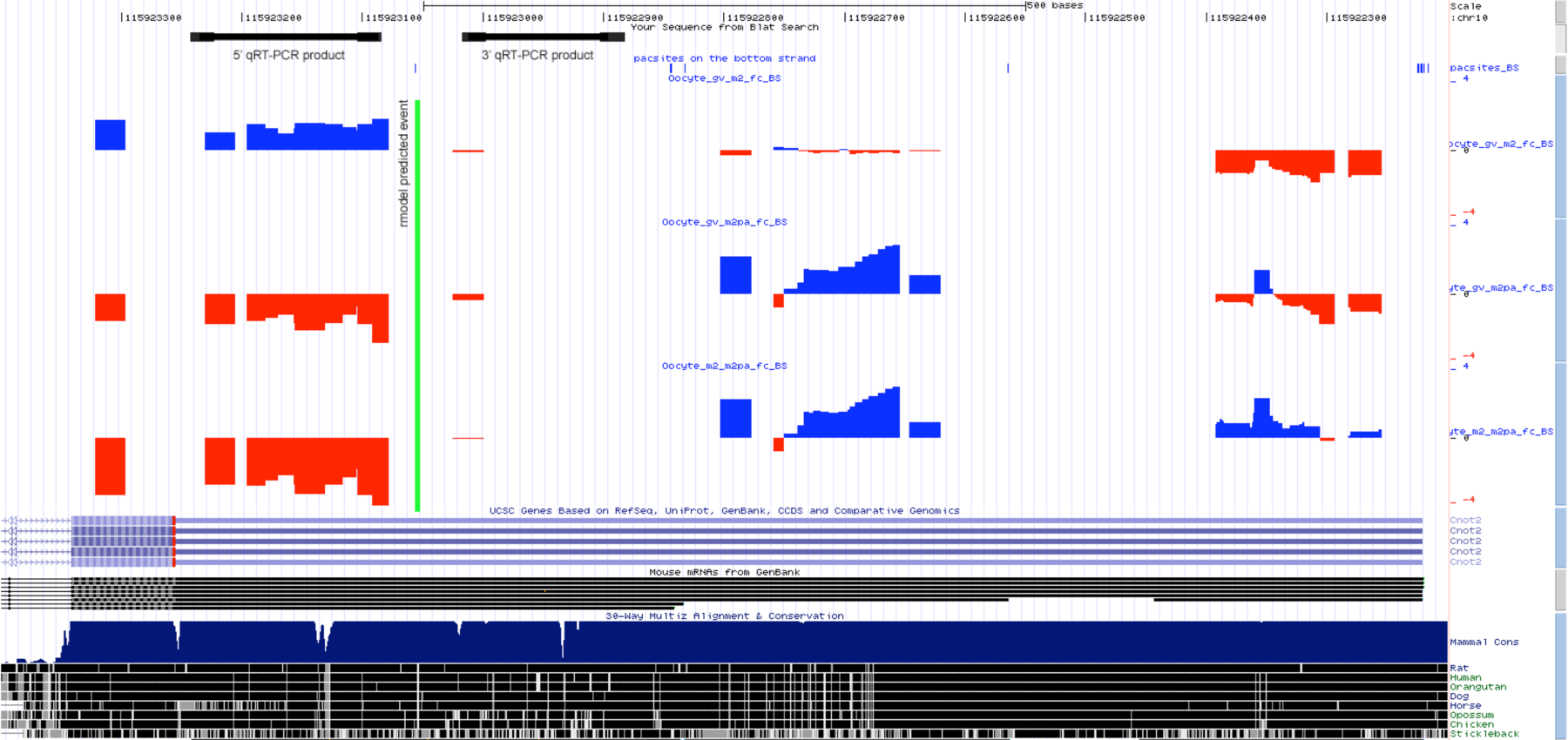

Supplement: Figure S8 — UCSC Genome Browser view of Cnot2 (MGI:1919318), showing the location of the rmodel predicted processing event (green line) and the location of the qRT-PCR products used to validate the processing change. (0.66 MB TIF) [file pone.0007479.s008.tif]

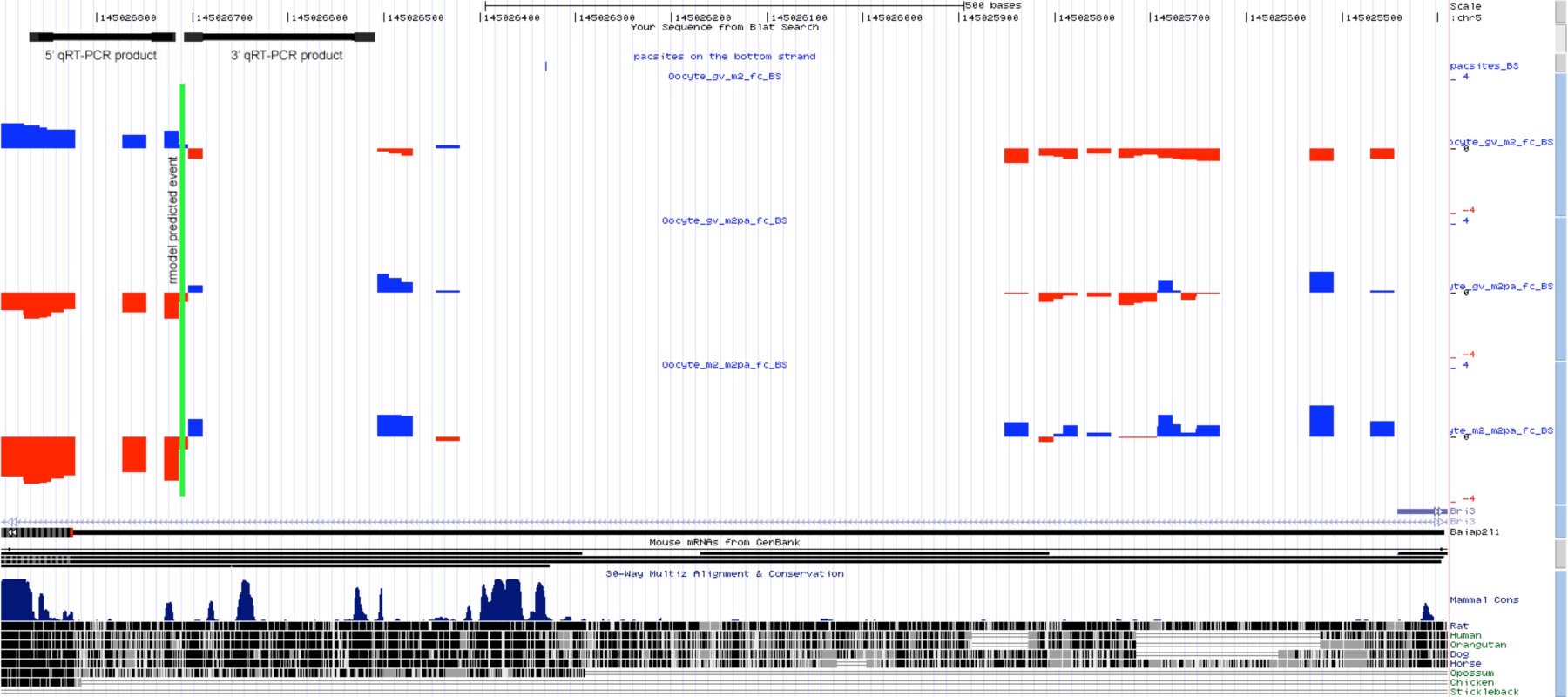

Supplement: Figure S9 — UCSC Genome Browser view of Baiap2l1 (MGI:1914148) showing the location of the rmodel predicted processing event (green line) and the location of the qRT-PCR products used to validate the processing change. (0.72 MB TIF) [file pone.0007479.s009.tif]

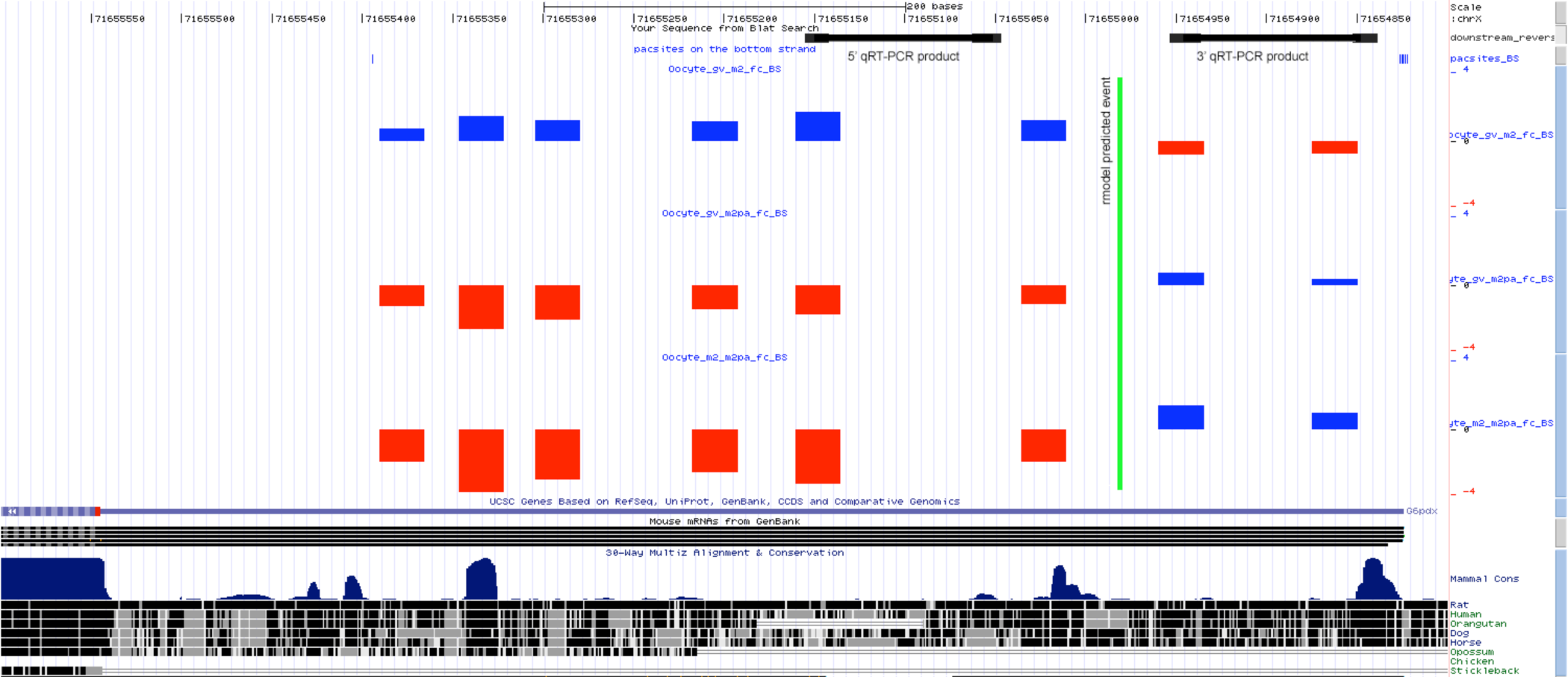

Supplement: Figure S10 — UCSC Genome Browser view of G6pdx (MGI:105979), showing the location of the rmodel predicted processing event (green line) and the location of the qRT-PCR products used to validate the processing change. (0.82 MB TIF) [file pone.0007479.s010.tif]

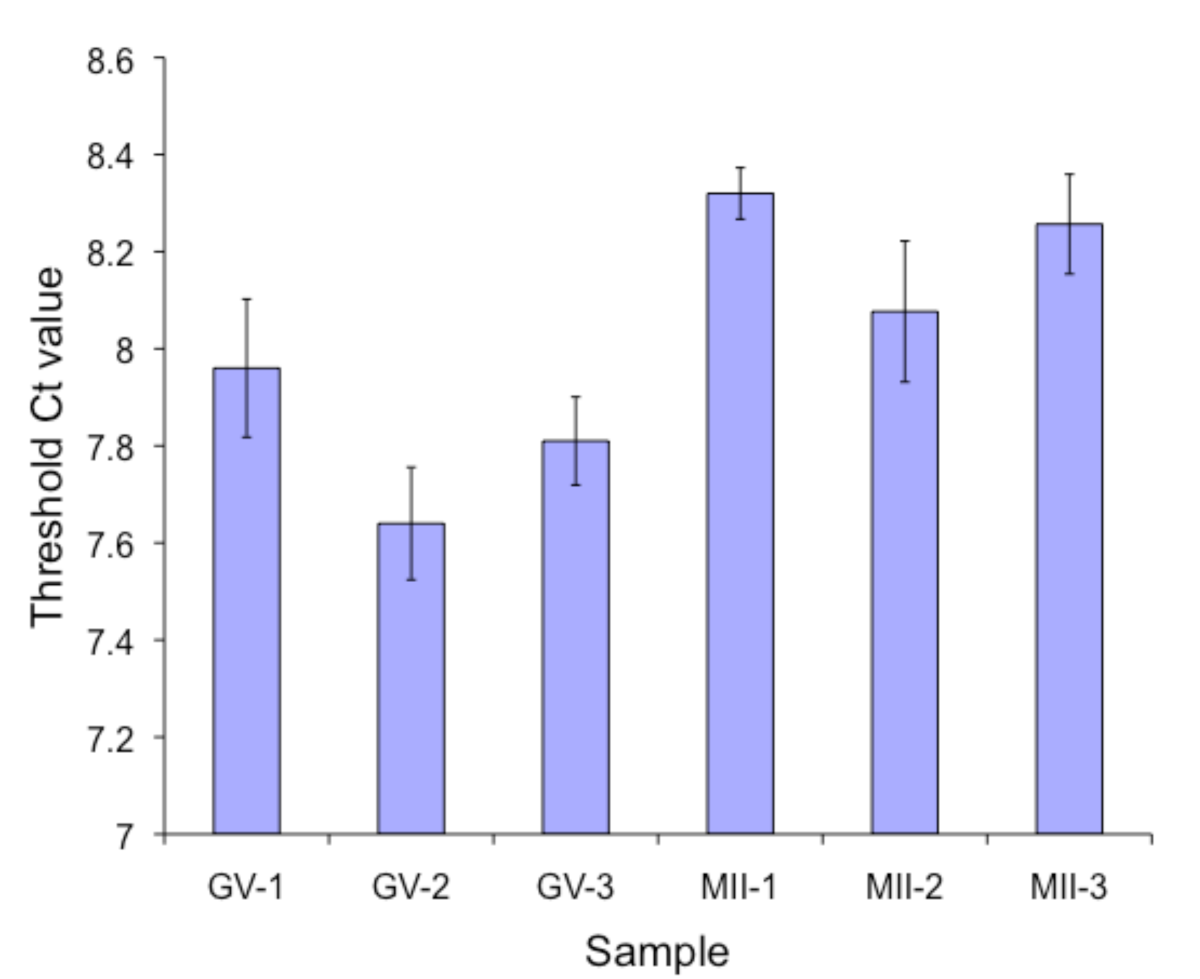

Supplement: Figure S11 — qRT-PCR results for the control Luciferase mRNA that was spiked into the oocyte cell extracts before RNA isolation and all subsequent steps. Bar heights represent the average Ct value obtained in three replicates of each sample. Error bars represent the standard error. The relatively low Ct value reflects the Luciferase transcript's dual role as carrier and control. (0.17 MB TIF) [file pone.0007479.s011.tif]
