# Supplementary material for: Probe-Level Analysis of Expression Microarrays Characterizes Isoform-Specific Degradation during Mouse Oocyte Maturation
Source: PLoS One. 2009 Oct 16;4(10):e7479. doi: 10.1371/journal.pone.0007479 (PMC2759528; doi:10.1371/journal.pone.0007479)
Supplement: Table S1 — Primers used in qRT-PCR validation of microarray results. (0.03 MB DOC) [file pone.0007479.s012.doc]

| gene | target | forward | reverse |
| --- | --- | --- | --- |
| *Mycbp* | 5’ region | CCGTGCATCTTCTGCTCTTT | GTGCAACCAGAACGCACTAA |
| *Mycbp* | 3' region | TGCTGCAGCTACCTTGATTG | TTGAGGGGAAAGAAATCACTG |
| *Atg5* | 5' region | GGAACCACCTTGAGTCAGGA | TCCTGTGTGTCTCAGCGAAG |
| *Atg5* | 3' region | TCTCAGGAGCTGTCACAGGA | GAAAGGAACGGGAGATTCAA |
| *Cnot2* | 5' region | TTTCTTGGGGTATGGCTGTC | TGGGCAGCACATAATTGGTA |
| *Cnot2* | 3' region | TTTGCCAGCAGACAGACTTG | TGAACAATGCAAATTTTGTGG |
| *G6pdx* | 5' region | TAGGCCTCAAAGGGACAATG | GGGCAGGTCCTCTCACATAG |
| *G6pdx* | 3' region | TCCCCTGACCAATTCCATAC | TTAATGGCAGGGTTGGGATA |
| *Baiap2l1* | 5' region | GCCAACAGTGACAAATGACC | CATCAGGCACTCTGCAATCT |
| *Baiap2l1* | 3' region | CCCCACTTGAGCAAATCAAT | CACCGACTATCCCTTATTCTGG |
