# Supplementary material for: Probe-Level Analysis of Expression Microarrays Characterizes Isoform-Specific Degradation during Mouse Oocyte Maturation
Source: PLoS One. 2009 Oct 16;4(10):e7479. doi: 10.1371/journal.pone.0007479 (PMC2759528; doi:10.1371/journal.pone.0007479)
Supplement: Text S1 — (0.04 MB DOC) [file pone.0007479.s013.doc]

## Supplemental Materials

# Probe-level analysis of expression microarrays characterizes isoform-specific degradation during mouse oocyte maturation

## Authors and Affiliations

### Jesse Salisbury^1^*, Keith W. Hutchison ^2^, Karen Wigglesworth^3^, John J. Eppig^3^, Joel H. Graber^1,3§^

^1^Functional Genomics PhD Program, University of Maine, Orono, Maine 04469-5708

^2^Department of Biochemistry, Microbiology and Molecular Biology, University of Maine, Orono Maine 04469-5735

^3^The Jackson Laboratory, 600 Main Street, Bar Harbor, Maine 04609

^§^Corresponding author

Email addresses:

JS: [jesse.salisbury@umit.maine.edu](mailto:jesse.salisbury@umit.maine.edu)

KWH: [keithh@maine.edu](mailto:keithh@maine.edu)

KW: [karen.wigglesworth@jax.org](mailto:karen.wigglesworth@jax.org)

JJE: [john.eppig@jax.org](mailto:john.eppig@jax.org)

JHG: [joel.graber@jax.org](mailto:joel.graber@jax.org)

**Supplemental Text**

**Comparison with Probe-Level Alternative Transcript Analysis (PLATA)**

PLATA [1], like rmodel, tests the segmentation of probe-level microarray data with a modified *t*-test, however, the normalization and pre-processing steps differ. The underlying similarity of the approaches led us to make a comparison of the methods.

**Remimplementation of PLATA in C++.** In order to compare rmodel analysis with PLATA, we created a C++ implementation of the algorithm, as described in the supplemental materials of the original manuscript [1]. Our reimplementation of the algorithm allowed us to test the equivalency of the statistical tests at all locations, rather than those that correspond only to known exon boundaries or processing sites. This decision did change PLATA slightly, as follows. In the original implementation, the probes were assigned to exons and portions of 3’-UTRs, and tests were made only on predetermined putative APA sites or exon boundaries. During the analysis for alternative activity, probes were removed if they did not meet a minimum expression threshold in at least one array. This resulted in the possibility of *t*-tests performed on regions with unequal numbers of probes, for instance if an APA site was flanked by a set of two valid probes upstream and a set of four valid probes downstream. In contrast, since we test all possible segmentations of the probeset, our PLATA implementation always tests equal numbers of probes on the two sides of a probeset. CEL files were processed into probesets according to our custom probeset for the Affymetrix Mouse 430 version 2 microarray. Background correction and normalization were performed with the *affy* analysis package (<http://www.biostat.jhsph.edu/~ririzarr/affy/>) for R (<http://www.R-project.org/>). The resulting probe-level data were analyzed with our implementation of PLATA.

We tested our version of PLATA on all possible segmentation points, using 2, 3, and 4 probes on either side of the probe, and varied the minimum background-corrected, normalized expression level required for a probe between 10 and 100. We also varied the minimum value for the variance within a probeset, as dicussed in the supplement of the original PLATA paper. The comparison between rmodel and PLATA was made as a correlation test between *t*-values for all putative segmentations at the same probeset and pair of probes. The *t*-value was used rather than the associated *p*-value, since rmodel uses a *t*-value threshold set by FDR rather than the standard *t*-test probability model.

The agreement between the t-values determined by the two methods was striking, with the Pearson correlation varying between 0.8 and 0.98 depending upon the exact parameters chosen for the analysis. Direct investigation of putative probesets that didn’t agree revealed that most were caused by differences in probes that were eliminated from consideration due to low expression level. Based on these analyses, we conclude that the mathematical aspects of PLATA and rmodel are essentially equivalent, and the principal difference lies in the selection of segmentations to test.

**References**

1. Sandberg, R., et al.*, Proliferating cells express mRNAs with shortened 3' untranslated regions and fewer microRNA target site*s. Science, 2008**. 3**20(5883): p. 1643-7.
